# An Unusual Presentation of a Lymphatic Malformation in an Adult: A Case Report

**DOI:** 10.5811/cpcem.2021.11.54618

**Published:** 2022-01-28

**Authors:** Marguerite Gilmore, Sharon H. Kim, Christopher M. McDowell

**Affiliations:** * College of the Holy Cross, Worchester, Massachusetts; † Southern Illinois University School of Medicine, Center for Clinical Research, Springfield, Illinois; ‡ Southern Illinois University School of Medicine, Department of Emergency Medicine, Springfield, Illinois

**Keywords:** lymphatic malformation, lymphangioma, case report

## Abstract

**Introduction:**

Patients commonly present with neck masses to the Emergency Department. The acute presentation of such a mass can be alarming to patients and their families. In this report we discuss a rare etiology of an acutely presenting neck mass in an adult.

**Case Report:**

We present a 19-year-old patient with an acute neck mass. The mass developed abruptly soon after initiation of a new upper body strength-training regimen. The patient’s history was unremarkable for any trauma or constitutional symptoms. Physical examination revealed the mass, which was diagnosed as a lymphatic malformation by imaging. Surgical removal was successful with pathology confirming the diagnosis.

**Conclusion:**

Lymphatic malformations, although rare, may present in adulthood. The acute presentation of a new mass, coupled with a lack of concerning constitutional symptoms, should increase the diagnostic suspicion of a lymphatic malformation.

## INTRODUCTION

Neck masses are a common presenting complaint among adult patients.^1^ The differential for these masses is vast but may be organized by acuity. Acute masses are more likely to be related to infection or trauma, whereas subacute are more likely to be malignant.^2^ An acute presentation without constitutional symptoms may indicate nonmalignant etiology. This case report highlights the importance of considering lymphatic malformation as a rare etiology of an acutely presenting neck mass. These malformations are typically detected and treated at birth or within the first two years of a child’s life and rarely present in adulthood.^3^,^4^ Although trauma has been implicated in rare presentations in adult cases, new strength-training regimens have not been previously reported as precipitating factors.^5^ In this case report, we discuss the presentation, physical examination, imaging studies, diagnosis, and surgical removal of an acute presentation of a lymphatic malformation in an adult.

## CASE REPORT

**Image 1:**
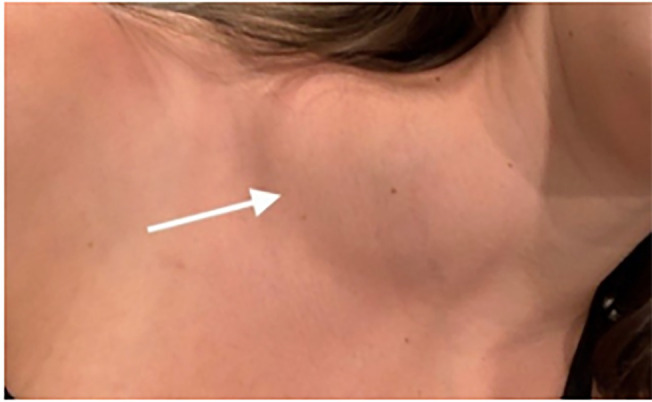
Image taken on same day patient found a new mass (arrow) on the right supraclavicular region of her neck.

An otherwise healthy 19-year-old female presented to an outlying emergency department (ED) on the same day that she noted an acutely developed mass on the distal right neck (Image 1). The patient’s past medical history was unremarkable, and she denied any constitutional symptoms such as fever, chills, night sweats, weight loss, or fatigue. There was no reported trauma. She denied difficulty swallowing but had noted slight shortness of breath. She was treated for a possible allergic reaction with diphenhydramine, but there was no response. An ultrasound was completed that showed a fluid collection in the supraclavicular area but no definitive diagnosis. The patient was instructed to follow up with her primary physician or return to the ED for any worsening symptoms.

The patient presented to our ED the following day as concern grew for the lack of definitive diagnosis, and she was evaluated again. Once again, she denied trauma, constitutional symptoms, or difficulty swallowing. She noted the mass was new and first discovered while showering the day prior. She had recently initiated a new strength-training regimen for her upcoming athletic season, which focused on upper body development 14 days prior to discovery of the neck mass. She was afebrile at 36.8° Celsius with blood pressure of 131/54 millimeters of mercury, pulse of 64 beats per minute, respiratory rate of 14 breaths a minute, and oxygen saturation of 100% on room air.

Physical examination revealed a large, easily palpable, nonerythematous, slightly tender mass that extended from eight centimeters (cm) proximal to the clavicle to the supraclavicular region. There was no palpable associated lymphadenopathy. Laboratory studies included complete blood count with white blood count of 10 thousand cells per cubic millimeter (K/uL) (reference range: 3.4–9.4 k/uL), platelets of 175 K/uL (140–410 K/uL), hemoglobin of 13.5 grams per deciliter (gm/dL) (12–16 gm/dL), erythrocyte sedimentation rate of 4 millimeters per hour (mm/hr) (0–20 mm/hr); thyroid-stimulation hormone of 1.15 microinternational units per milliliter (μIU/ML) (0.45–5.33 μIU/ML); and mononucleosis test negative.

Computed tomography (CT) of the neck and soft tissue revealed a large cystic collection along the right neck deep to the sternocleidomastoid muscle and extending inferiorly into the supraclavicular region with two smaller, adjacent cystic collections. No aerodigestive tract mass or cervical lymphadenopathy was noted. The primary consideration was a large lymphatic malformation. Outpatient otolaryngology follow-up was arranged after they reviewed the CT results and concurred with the presumed lymphatic malformation diagnosis.

**CPC-EM Capsule**

What do we already know about this clinical entity?

*Lymphatic malformations are usually diagnosed within the first two years of life*.

What makes this presentation of disease reportable?

*This was an acute presentation of a lymphatic malformation in an adult which is unusual in both acuteness and age of the patient*.

What is the major learning point?

*Lymphatic malformation should be considered in the differential diagnosis of an acutely presenting neck mass regardless of age*.

How might this improve emergency medicine practice?

*Emergency physicians can provide reassurance that acutely presenting masses lacking constitutional symptoms, recent illness, or associated lymphadenopathy may portend a better prognosis*.

**Image 2:**
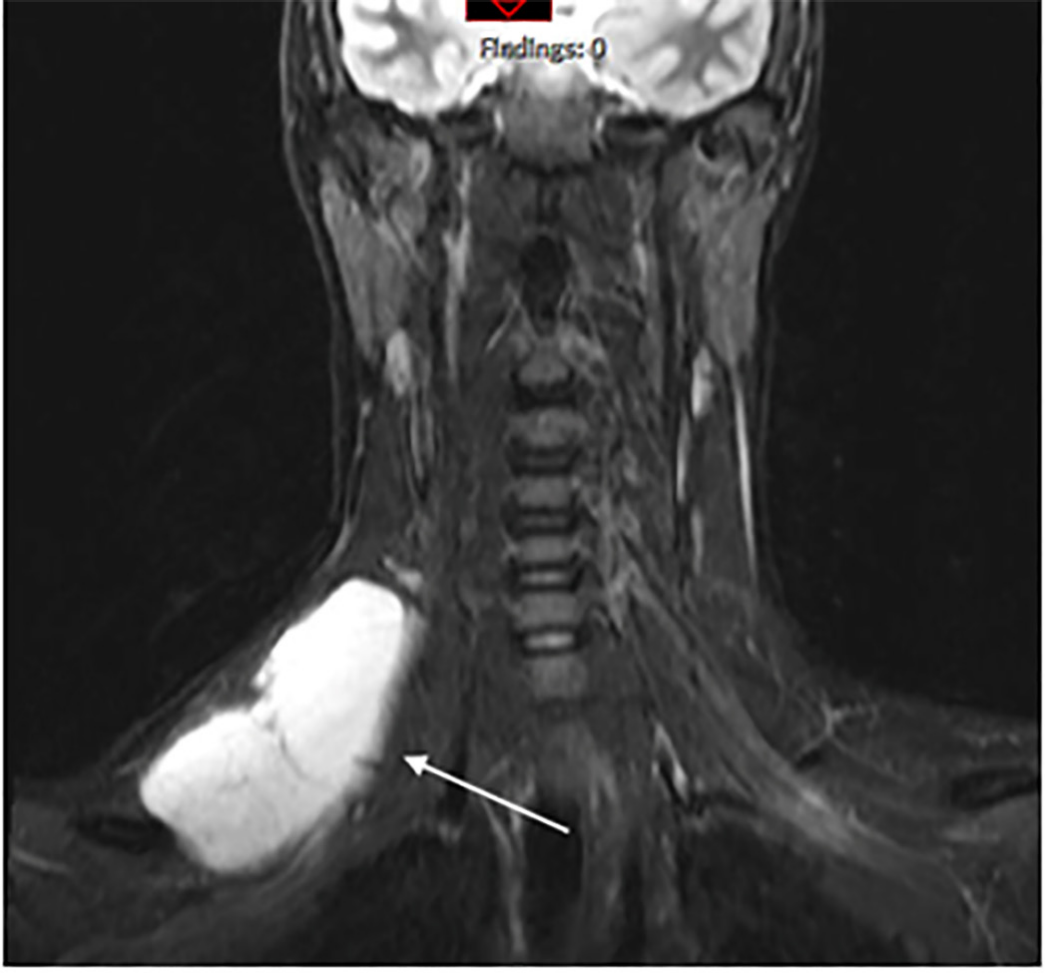
Coronal view T2 short T1 inversion recovery magnetic resonance image with arrow depicting hyperintense supraclavicular lesion consistent with lymphatic malformation.

The patient subsequently underwent magnetic resonance imaging (MRI) that was consistent with a lymphatic malformation (Image 2). Approximately three weeks after discovery, otolaryngology removed the mass, and pathology revealed a 68-gram 10.5 × 5.5 × 3.5 cm mass that was diagnosed as a lymphangioma.

After recovering from the surgery, the patient began physical therapy for her shoulder. At 10 weeks post-surgery, the patient was continuing with physical therapy and able to return to her collegiate crew participation.

## DISCUSSION

The differential for neck masses can be differentiated by acuity of presentation. Acute masses are most often infectious or traumatic. Subacute or chronic masses are more often considered to be malignant or related to other chronic illness (Table).^11^

Lymphatic malformations are uncommon, benign masses that result from abnormal lymphatic system development. The preferred term lymphatic malformation encompasses cystic hygromas, lymphangiomas, cavernous lymphangiomas, cystic lymphangiomas, and lymphangioma circumscriptum. In the majority of cases, these masses are recognized by two years of age, where prevalence is believed to be approximately 1:4000 live births.^6^ These abnormalities are most commonly found in the head or neck but can be seen throughout the body’s lymphatic system.^7^ These presentations are so rare in adulthood that the prevalence is not well defined.^8^ Although precipitation by trauma has been reported in very rare circumstances, we are unaware of prior presentations due to new exercise regimens.^5^,^9^,^10^ In our case, the patient had initiated a new upper body strength-training regimen about 14 days prior to mass discovery. These sessions included a 20–30 minute arm circuit that focused on arm flies, shoulder presses, and push-ups. The patient denied any new trauma or noted injuries during strength training.

Diagnosis of lymphatic malformations usually requires advanced imaging. Determining which imaging modality to choose can be difficult for the emergency physician. Ultrasound can determine cystic structures but lacks the ability to reliably establish mass etiology.^11^ Computed tomography and MRI multiplanar images reveal better identifying characteristics. In addition, these images are especially helpful in surgical planning.^11^ Histopathological assessment is necessary to confirm diagnosis in adults.^12^ In the absence of dysphagia or breathing difficulty, a stepwise diagnostic progression would be reasonable. This may include ED referral to the primary physician or otolaryngology for more definitive imaging.

Treatment options for lymphatic malformations include percutaneous drainage, surgery, sclerotherapy, laser therapy, and radiofrequency ablation.^3^ Aspiration can be helpful in diagnosis but often does not prevent recurrence. Previous cases suggest surgical excision as the preferred treatment modality in adults to mitigate tumor recurrence.^12^,^13^ Decisions regarding treatment strategies will depend on location and associated symptoms as well. For instance, patients suffering from dysphagia, dyspnea, or other vital structures at risk often require surgery. Conversely, complete resection may not be possible due to localization and microvasculature of the mass near essential organs, in which case alternative methods must be considered.^9^

## CONCLUSION

We report a case of a lymphatic malformation that presented acutely as a new neck mass in an adult. Rapidly developed new-onset masses should be evaluated as possible lymphatic malformations. The lack of constitutional symptoms and rapid onset make malignant neoplasm less likely. Advanced CT and MRI imaging led to the diagnosis and helped to prepare for successful surgical excision.

## Figures and Tables

**Table:** Neck mass etiologies by onset.^2^

Presentation	Common	Uncommon
Acute	Viral upper respiratory infection Reactive lymphadenopathy: Epstein-Barr virus Cytomegalovirus Toxoplasmosis	Human immunodeficiency virus *Mycobacterium tuberculosis* Hematoma Acute sialoadenitis Pseudoaneurysm Trauma
Subacute (weeks to months)	Cancer: Hodgkin & non-Hodgkin lymphoma Human papillomavirus-related Squamous cell carcinoma Metastatic cancer Parotid tumor	Amyloidosis Sarcoidosis Sjogren syndrome Branchial cleft cyst
